# Material decomposition using iodine quantification on spectral CT for characterising nodules in the cirrhotic liver: a retrospective study

**DOI:** 10.1186/s41747-021-00220-6

**Published:** 2021-05-28

**Authors:** Shalini Thapar Laroia, Komal Yadav, Senthil Kumar, Archana Rastogi, Guresh Kumar, Shiv Kumar Sarin

**Affiliations:** 1 Department of Radiology, Institute of Liver and Biliary Sciences, Sector D-1, Vasant Kunj, New Delhi, 110070 India; 2 Department of HPB Surgery and Liver Transplantation, Institute of Liver & Biliary Sciences, Sector D-1, Vasant Kunj, New Delhi, 110070 India; 3 Department of Clinical and Hepato-pathology, Institute of Liver and Biliary Sciences, Sector D-1, Vasant Kunj, New Delhi, 110070 India; 4 Department of Biostatistics and Research, Institute of Liver & Biliary Sciences, Sector D-1, Vasant Kunj, New Delhi, 110070 India; 5 Department of Hepatology, Institute of Liver & Biliary Sciences, Sector D-1, Vasant Kunj, New Delhi, 110 070 India

**Keywords:** Carcinoma (hepatocellular), Iodine, Liver cirrhosis, Liver neoplasms, Tomography (x-ray, computed)

## Abstract

**Background:**

There is limited scientific evidence on the potential of spectral computed tomography (SCT) for differentiation of nodules in the cirrhotic liver. We aimed to assess SCT-generated material density (MD) parameters for nodule characterisation in cirrhosis.

**Methods:**

Dynamic dual-energy SCT scans of cirrhotic patients performed over 3 years were retrospectively reviewed. They were classified as hepatocellular carcinoma (HCC), regenerative or indeterminate, according to the European Association for the Study of the Liver criteria. MD maps were generated to calculate the area under the curve (AUC) and cutoff values to discriminate these nodules in the hepatic arterial phase (HAP) and portal venous phase (PVP). MD maps included iodine concentration density (ICD) of the liver and nodule, lesion-to-normal liver ICD ratio (LNR) and difference in nodule ICD between HAP and PVP.

**Results:**

Three hundred thirty nodules belonging to 300 patients (age 53.0 ± 12.7 years, mean ± standard deviation) were analysed at SCT (size 2.3 ± 0.8 cm, mean ± SD). One hundred thirty-three (40.3%) nodules were classified as HCC, 147 (44.5%) as regenerative and 50 (15.2%) as indeterminate. On histopathology, 136 (41.2%) nodules were classified as HCC, 183 (55.5%) as regenerative and 11 (3.3%) as dysplastic. All MD parameters on HAP and the nodule  difference in ICD could discriminate pathologically proven HCC or potentially malignant nodules from regenerative nodules (*p* < 0.001). The AUC was 82.4% with a cutoff > 15.5 mg/mL for nodule ICD, 81.3% > 1.8 for LNR-HAP and 81.3% for difference in ICD > 3.5 mg/mL.

**Conclusion:**

SCT-generated MD parameters are viable diagnostic tools for differentiating malignant or potentially malignant from benign nodules in the cirrhotic liver.

**Supplementary Information:**

The online version contains supplementary material available at 10.1186/s41747-021-00220-6.

## Key points

- Spectral computed tomography-generated material density values provide a viable quantitative imaging tool.
- Material density parameters can discriminate malignant from benign cirrhotic nodules.
- This model offers an add-on diagnostic tool for the characterisation of cirrhotic nodules.

## Background

End-stage nodular cirrhosis is the final pathological entity of many different progressive liver diseases [1, 2]. Cirrhotic nodules can be classified as benign (regenerative), indeterminate (potentially malignant/dysplastic) or malignant (hepatocellular carcinoma, HCC) [3]. Regenerative nodules are considered ‘benign’ due to a lack of phenotypically abnormal cells but are known to contain genomically atypical cells [4–6]. Dysplastic nodules containing both normal and dysplastic hepatocytes are considered precursors to HCC. Inconclusive imaging features such as arterial hypervascularity without subsequent washout or hypodensity on delayed phase without overt arterial enhancement with nodule size > 1 cm may be used to identify indeterminate nodules at computed tomography (CT) [7–13].

The main radiological system to classify cirrhotic nodules at CT and MRI is the Liver Imaging Reporting and Data System (LI-RADS) [14]. According to the LI-RADS, all cirrhotic nodules have the potential to be HCC, ranging from least likely, namely LI-RADS category 1 (definitely benign), to most, LI-RADS category 5 (definitely malignant) [14]. Mid-spectrum categories such as LI-RADS 3 or 4 fall into the indeterminate zone of diagnostic suspicion, *i.e.*, they do not represent definite HCC nor benign regenerative nodules [15]. Such lesions, especially nodules between 1 and 2 cm in size, are often found to be dysplastic or well-differentiated HCC on pathological examination [16–19]. The most recent LI-RADS-based guidelines have shown a sensitivity of 89.6%, a specificity of 81.2% and an overall diagnostic accuracy of 88.0% for HCC lesions > 2 cm [20].

The advent of spectral computed tomography (SCT) and its ability to generate synchronous datasets of polychromatic and monochromatic images with material density (MD) maps has enabled radiologists to experiment beyond the conventional enhancement pattern-based algorithms, which visually assess contrast uptake, retention and washout [21–25]. The most recent upgrades in CT hardware and software allowed the use of SCT for tissue characterisation using MD maps [26]. In fact, at SCT, images from two distinct x-ray spectra at 70–80 kVp (low energy) and 120–140 kVp (high energy) can be exploited to visualise hypervascular lesions with better conspicuity on MD datasets [25, 27, 28]. MD images are used to calculate the iodine concentration density (ICD) of the lesion, measured in milligrams per milliliter along with other parameters [29–32].

Most SCT liver studies have investigated the diagnosis and differentiation of HCC from benign hypervascular lesions such as haemangioma, fibronodular hyperplasia or angiomyolipoma in noncirrhotic populations [33–36]. However, there is a lack in existing scientific knowledge regarding the potential of SCT for the overall differentiation of nodules in the cirrhotic liver [37–39].

Thus, the aim of this study was to assess the diagnostic performance of SCT for the discrimination of malignant (HCC), potentially malignant (indeterminate) and benign (regenerative) nodules in cirrhosis patients.

## Methods

### Population

This single-centre retrospective study was approved by the institutional ethics board, and the requirement for informed consent was waived. We screened SCT scans of 380 cirrhotic patients performed from January 2016 to June 2019. SCT scans were obtained by searching the hospital and radiology database for the cirrhotic population who underwent pre-transplant and surveillance CT scans. For inclusion in the study, patients who underwent pre-transplant CT, as per institute protocol (*n* = 300), and those who were found to have liver nodules on CT, who subsequently underwent biopsy (*n* = 20), were included. Patients with identifiable nodules on SCT who did not undergo liver transplantation or follow-up with biopsy (*n* = 80) were excluded.

**Fig. 1:**
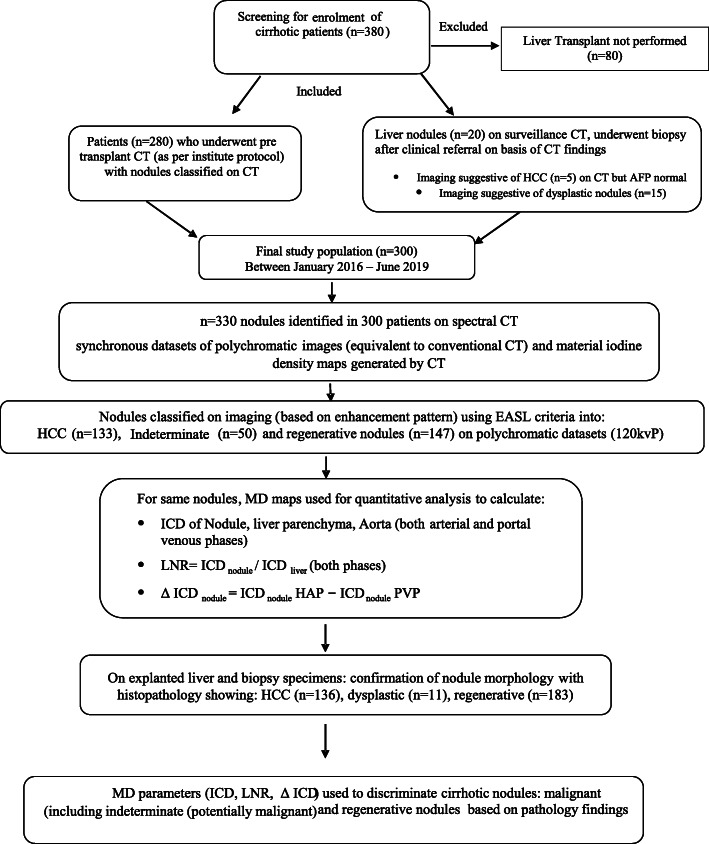
Flowchart of the study design and workflow. *AFP* Alpha-fetoprotein, *CT* Computed tomography, *EASL* European Association for the Study of the Liver, *HCC* Hepatocellular carcinoma, *ICD* Iodine concentration density. *LNR* = ICD_nodule_/ICD_liver_, ICD difference of nodule (ΔICD_nodule_) = ICD_nodule_ HAP minus ICD_nodule_ PVP

A total of 330 nodules from 300 selected SCT studies were independently evaluated by two observers with more than 4 years of experience in liver imaging. Conflicting observations between the two reviewers were re-evaluated at a consensus meeting, and a final decision was taken in the presence of a third reviewer. The workflow of the study is shown in Fig. 1.

### SCT protocol

Scans that were performed as a triple-phase study on the single-source, dual-energy, 64-slice CT scanner (Discovery CT 750 HD, General Electric Healthcare, USA) with fast kVp switching between 80 and 140 kVp were included. Scanning protocol and technique are summarised in Table 1.

**Table 1:** Computed tomography protocol

Mode	Supine position Spectral imaging mode Fast tube voltage switching between 80 and 140 kVp Craniocaudal direction
Collimation thickness	0.625 mm (64 detectors)
Slice thickness	5 mm
Reconstruction parameters	1-mm thickness, 1-mm interval
Tube current	600 mA
Rotation speed	0.6 s
Helical pitch	0.983
Reconstruction	Adaptive statistical iterative reconstruction (ASIR)
Contrast agent	Iomeprol, Bracco, Konstanz, Germany (400 mgI/mL)
Contrast agent dose and rate	1.5 mL/kg body weight, at the rate of 4 mL/s
Saline bolus and rate	50 mL at the rate of 4 mL/s
Scan delay	Automated triggering (threshold of 100 HU in the aorta)

### Image analysis

**Fig. 2:**
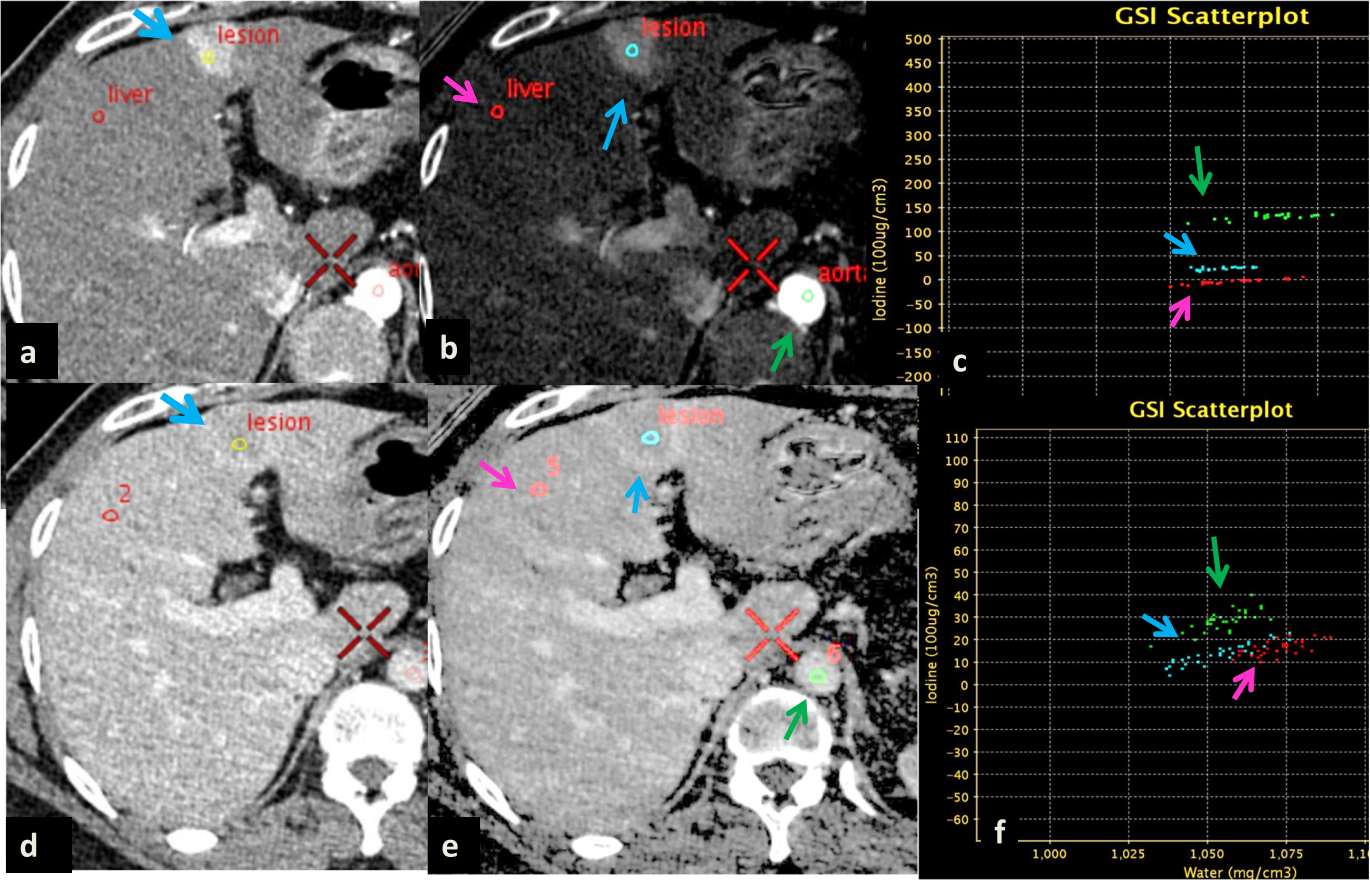
Spectral computed tomography (SCT) of a patient with cirrhosis and hepatocellular carcinoma depicting polychromatic, material density (MD) map, and scatter plots. **a** Arterial phase axial polychromatic image showing well-defined subcapsular hypervascular lesion (HCC), measuring 2.4 cm in segment IV (blue arrow). **b** Iodine density map showing ROI (blue circle) within lesion to calculate ICD_nodule_ HAP (blue arrow), ROI in the liver (red circle) to measure ICD_liver_ HAP (pink arrow), and ICD_aorta_ (green arrow). **c** Scatter plot depicting the highest iodine density value of the aorta (green arrow), lesion iodine density (blue arrow) less than the aorta but more than the background liver (pink arrow). **d** Portal venous phase polychromatic image showing segment IV lesion depicting washout (yellow circle) with capsular enhancement (blue arrow). **e** Corresponding iodine density map showing ROI within lesion (blue circle) to calculate ICD_nodule_ PVP, ICD_aorta_ PVP (green circle), and ICD_liver_ PVP (pink circle). **f** Scatter plot depicting the highest ICD_aorta_ PVP (green arrow), ICD_nodule_ PVP (blue arrow) showing a decreasing trend of iodine density compared to the background liver (pink arrow)

**Fig. 3:**
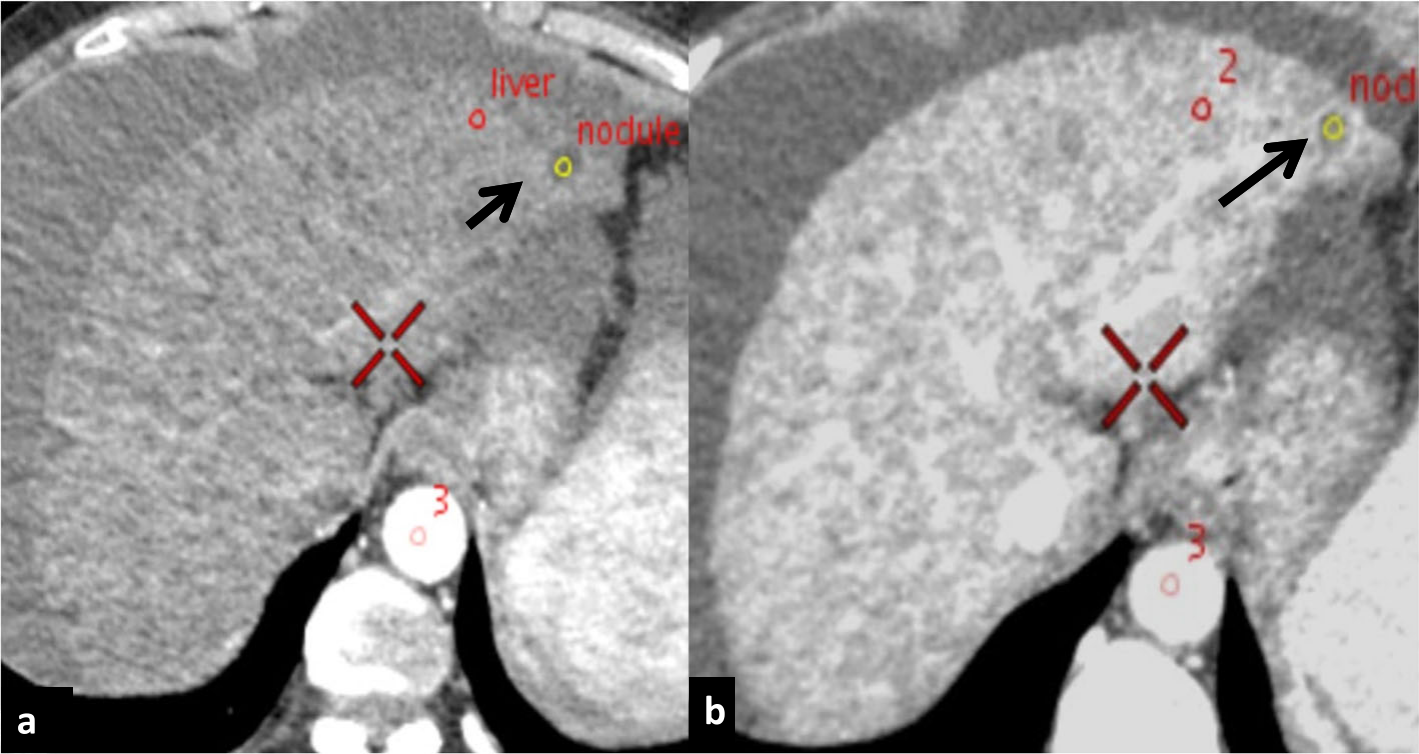
Computed tomography of a patient with underlying cirrhosis depicting ‘regenerative’ nodule. **a** Polychromatic axial image on HAP showing a well-defined subcapsular non-enhancing lesion (yellow ROI), in segment II (black arrow). **b** Polychromatic PVP image shows persistent non-enhancement of the same lesion (yellow ROI) in segment II (black arrow)

The polychromatic image sets with 120 kVp photon energy, which are analogous to the conventional CT images, were used to classify cirrhotic nodules into HCC, indeterminate and regenerative based on enhancement pattern of lesions on hepatic arterial phase (HAP) and portal venous phase (PVP) (Fig. 2). Nodules with HAP hypervascularity and subsequent washout on PVP or delayed phase were classified as HCC [40]. Nodules that did not show classical features of HCC (neither hypervascular nor displaying washout) were classified as ‘regenerative’ (non-HCC by EASL criterion) (Fig. 3) [17]. Since we excluded all other liver lesions at the beginning of the study, benign lesions were synonymous for regenerative nodules for this study. Nodules with ambiguous enhancement, such as isolated arterial hypervascularity or exhibition of washout or capsule formation on PVP without arterial enhancement, were grouped as ‘indeterminate’ nodules [41].

The MD images generated by SCT were viewed and analysed with material decomposition and projection-based software on the workstation (CTAW software 4.4, General Electric Healthcare, USA), with a standard soft-tissue display window (preset at 40 HU and width 400 HU). Images were reviewed at a standard reconstruction kernel on HAP and PVP.

Iodine density of the nodule (ICD_nodule_), liver parenchyma (ICD_liver_) and aorta (ICD_aorta_) were measured in milligrams per milliliter on HAP and PVP with the help of three discrete circular regions of interest (ROIs) of equal area, size and shape, carefully placed in the maximum enhancing portion of each structure, at the same level in the abdomen, as shown in Fig. 2. The average number of pixels in each ROI was 400 (range 310–560). Three parameters were calculated using the above values: lesion-to-normal liver ratio (LNR = ICD_nodule_/ICD_liver_), normalised iodine concentration (NIC = ICD_nodule_/ICD_aorta_) and difference in ICD (ΔICD = ICD_nodule_ HAP − ICD_nodule_ PVP). The NIC was measured as a baseline reference for optimum enhancement, to minimise variations among patients.

**Fig. 4:**
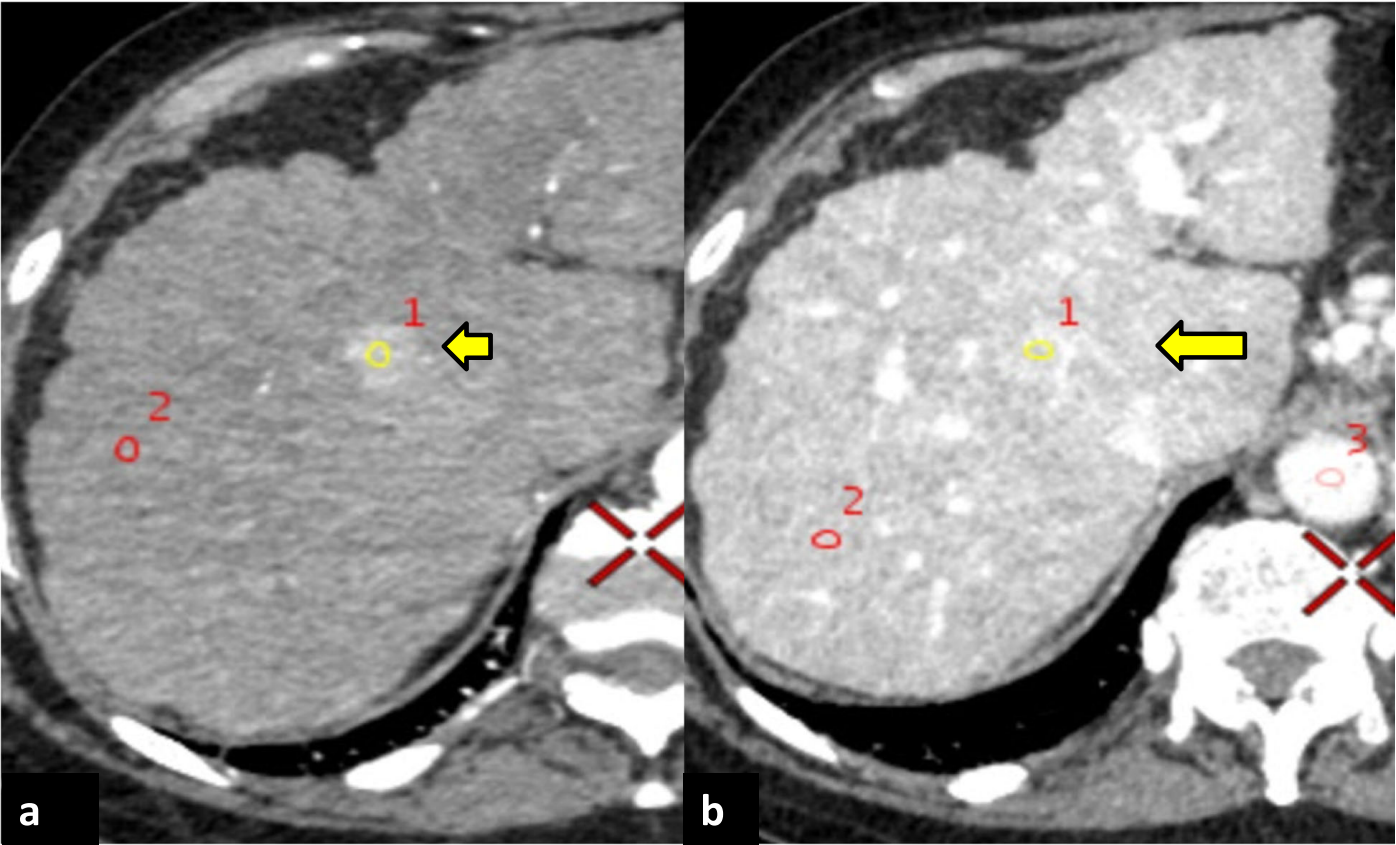
Computed tomography of a patient with underlying cirrhosis depicting an ‘indeterminate’ nodule. **a** Polychromatic axial image on HAP depicting a hypervascular nodule (yellow ROI), in segment VIII (yellow arrow). **b** Polychromatic axial image on PVP of the same nodule shows persistent enhancement without overt washout (yellow ROI) in segment VIII (yellow arrow)

For MD parameter analysis, nodules were organised into two broad groups: malignant (HCC), also including potentially malignant (indeterminate) nodules, *versus* benign (regenerative) nodules owing to the limitation of pathological confirmation of dysplastic nodules and the significance of their stepwise progression to frank HCC [8, 10, 11] (Fig. 4).

Serum alpha-fetoprotein (AFP) values were recorded for all patients at the time the scan was performed, with observer blinding and used for statistical and comparative data analysis. Histopathology results of explant liver specimens (310 nodules) and nodule biopsy (20 nodules) were used as a reference standard.

### Histopathological analysis

Percutaneous ultrasound-guided liver biopsy was performed using an 18-gauge cutting needle. Explant and biopsy specimens were routinely processed for systematic histological evaluation. All biopsies were read by a liver pathologist with 15 years of experience who was blinded to imaging results.

Regenerative nodules were classified on basis of features of a hyperplastic or a hepatocellular nodule at low magnification, without distinct cytoarchitectural features from the extralesional cirrhotic background, intralesional 2 cell-thick hepatocyte plates and unconnected lesion portal tracts [8]. Dysplastic nodules were characterised by architectural abnormalities such as 2 or 3 cell-thick hepatocyte plates, low-grade cytological atypia, nuclear crowding, inconstant detection of map-like clonal growth or nodule-in-nodule appearance, and inconstant increase in the number of unpaired arterioles which was confirmed by smooth muscle actin immunostaining. Progressive sinusoidal capillarisation was detected by CD34 immunostaining. Distinction from well-differentiated HCC is known to be challenging [9–11, 16]. A diagnosis of HCC was finalised on seeing architectural abnormalities such as nuclear crowding, three or more cell-thick hepatocyte plates, relative cell monomorphism, presence of multiple microacinar structures with or without bile plugs, an abnormally high number of muscularised unpaired arterioles, capillarised vessels and infiltration of the portal tract, fibrous septa, or veins by single hepatocytes [9–11, 16].

### Statistical analysis

Continuous data were presented as mean ± standard deviation (SD) or median with interquartile range (IQR) according to data normality. The comparison was made using a one-way analysis of variance (ANOVA) test, followed by a post hoc test by the Bonferroni method. Categorical data were reported as numbers and frequencies (%) and compared by using the *χ*^2^ test or Fisher exact test as applicable. The agreement between two observers was assessed using kappa (*κ*) statistics.

The comparison between malignant or potentially malignant (HCC and indeterminate) and benign (regenerative) nodules for comparing and analysing the significance of MD parameters on SCT was computed using the Mann-Whitney *U* test or independent *t*-test as appropriate. Sensitivity, specificity, positive predictive values and negative predictive values were calculated with their 95% confidence intervals (CI), according to the binomial distribution. The area under the curve (AUC) at receiver operating characteristic (ROC) analysis was also calculated*.* The quantification of effect (odds ratio, OR) was based on the nominal logistic regression where imaging discrimination of more than two nodules, *i.e.*, HCC, indeterminate/dysplastic or regenerative, was calculated. The logistic regression analysis was used to quantify the effect and to derive cutoff values for malignant or potentially malignant and benign nodules. All analyses were performed using SPSS statistical software (version 22, IBM, USA). A *p* value less than 0.05 was considered to indicate statistical significance.

## Results

The mean size of 330 analysed nodules was 2.3 ± 0.8 cm. Such 330 nodules were found in 300 cirrhotic patients of which 280 were pre-transplant candidates and 20 were under surveillance. The majority of patients (*n* = 266; 89%) were men; mean patients’ age was 53.2 ± 12.7 years. The interobserver agreement between the two radiologists gave a *κ* value of 0.95. All details of imaging diagnosis and pathology correlation are shown in Table 2 and Supplementary Table 3. Enhancement characteristics of nodules are detailed in Table 3.

**Table 2:** Characteristics of patients with CT diagnosis of liver nodules and histopathology correlation

Characteristics	Total nodules 330	HCC on CT 133 (40.3%)	Indeterminate on CT 50 (15.2%)	Regenerative on CT 147 (44.5%)	*p*-value
Age (years, mean ± SD)	53.2 ± 12.7	58.6 ± 11.6	56.7 ± 10	47.1 ± 11.8	< 0.001
Gender (male/female)	266/64	108/25	38/12	120/27	0.667
Log AFP (ng/mL, mean ± SD)	2.63 ± 3.1	3.5 ± 2.3	3.01 ± 2.4	1.7 ± 1.1	< 0.001
Nodule size (cm, median (IQR))	1.7 (1.1–2.1)	2.1 (1.9–5.0)	1.4 (1.2–1.6)	1.2 (1–1.8)	< 0.001
Increased AFP (> 8.5 ng/mL) (%)	138 (41.8)	84 (63.1)	31 (62)	23 (15.6)	< 0.001
Nodules classified according to size (largest diameter) on CT					
< 1 cm (% of total nodules)	29 (8.8)	0 (0)	2 (4)	27 (18.5)	< 0.001
1–2 cm (% of total nodules )	198 (60)	48 (36)	44 (88)	106 (72)	
> 2 cm (% of total nodules)	103 (31.2)	85 (63.9)	4 (8)	14 (9.5)	
CT histopathology correlation					
Histopathology classification (%)	HCC 136 (41.2%)		Dysplastic 11 (3.3%)	Regenerative 183 (55.5%)	
True positive on CT	129		7	141	
True negatives on CT	190		276	141	
False positive on CT	4		43	6	
False negative on CT	7		4	42	
Sensitivity % (95% CI)	94.9 (89.8–97.5)		63.6 (35.4–84.8)	77.0 (70.4–82.5)	
Specificity % (95% CI)	97.9 (94.8–99.2)		86.5 (82.3–89.8)	95.9 (91.4–98.1)	
PPV % (95% CI)	96.9 (92.5–98.8)		14 (6.9–6.2)	95.9 (91.4–98.1)	
NPV % (95% CI)	96.5 (92.9–98.3)		98.6 (96.4–99.44)	77.1 (70.4–82.5)	
Accuracy % (95% CI)	96.7 (94.1–98.1)		85.8 (81.6–89.1)	85.5 (81.2–88.9)	

*AFP* Alpha-fetoprotein, *CT* Computed tomography, *CI* Confidence interval, *IQR* Interquartile range, *SD* Standard deviation, *NPV* Negative predictive value, *PPV* Positive predictive value

**Table 3:** Enhancement pattern of nodules on computed tomography

Enhancement pattern on different phases		Total nodules 330	HCC 133	Indeterminate 50	Regenerative 147	*p*-value
Hypervascular	HAP (%)	124 (37.6)	98 (73.7)	24 (48)	2 (1.4)	< 0.001
Hypoattenuating	HAP (%)	159 (48.2)	8 (6)	11 (22)	140 (95.2)	< 0.001
	PVP (%)	320 (96.9)	131 (98.5)	46 (92)	143 (97.3)	0.084
Mild or subtle enhancement	HAP (%)	47 (14.2)	27 (20.3)	15 (30)	5 (3.4)	< 0.001
	PVP (%)	10 (3)	2 (1.5)	4 (8)	4 (2.7)	0.084
Washout present	*n* (%)	166 (50.3)	125 (93.9)	40 (80)	1 (0.7)	< 0.001

On HAP, 124/330 (38%) nodules were hypervascular with 98/124 (79%) demonstrating subsequent washout, characteristic of HCC (Table 3). Mild enhancement on HAP was present in 47/330 (14%) nodules with 27/47 (57%) of these subsequently showing washout. These were also classified as HCC (Table 3). Some other nodules, 24/124 (19%) which showed hypervascularity, but inadequate washout, were categorised as indeterminate nodules.

A total of 159/330 (48%) nodules were non-enhancing on HAP, 140/159 (88%) of which showed consistent hypoattenuation on PVP as well. Based on this pattern, these were classified as regenerative nodules (Table 3).

A total of 166 nodules demonstrated contrast washout. Most of them, 125/166 (75%), were classified as HCC; few, 40/166 (24%), were diagnosed as indeterminate, due to the absence of classical arterial hypervascularity; and one nodule was classified as regenerative (1%) (Table 3). The details of enhancement, with reference to nodule size, have been enumerated in Supplementary Table 1.

A total of 133/330 (40.3%) nodules were classified as HCC, of which 129 were confirmed on pathology. Among the remaining false-positive HCC’s, one nodule was dysplastic and three regenerative. Thus, for diagnosis of HCC, CT demonstrated a sensitivity of 94.9% (95% CI 89.8–97.5%) and a PPV of 96.9% (95% CI 92.5–98.8) (Table 2).

A total of 147/330 (44.5%) nodules were classified as ‘regenerative’ on CT whereas 183/330 (55.5%) nodules were confirmed ‘regenerative’ on pathology (Table 2). On CT, 37 biopsy-proven regenerative nodules (measuring 1–2 cm) and two nodules (measuring < 1 cm) were categorised as ‘indeterminate’. Three nodules (size 1–2 cm) were also inadvertently classified as HCC. CT thus demonstrated a sensitivity of 77% (95% CI 70.4–82.5%) and PPV of 95.9% (95% CI 91.4–98.1%) for diagnosis of regenerative nodules.

CT observations correctly classified 97% (95% CI 94.1–98.1%) of HCC and 85.5% (95% CI 81.2–88.9%) of regenerative nodules at pathology (Table 2).

A total of 50/330 (15.2%) lesions were classified as indeterminate nodules on CT; on pathology, 39/50 (78%) of these were regenerative and 4/50 (8%) were HCC, whereas only 5/50 (10%) were dysplastic nodules (Table 3). In comparison with pathology, CT demonstrated a sensitivity of 63.6% (95% CI 35.4–84.8%) and a PPV of 14% (95% CI 6.9–6.2%) for indeterminate nodules.

MD datasets from SCT were subsequently analysed to distinguish benign and malignant (including potentially malignant) cirrhotic nodules using MD parameters. They demonstrated an overall higher diagnostic sensitivity of 95.9% (95% CI 91.4–98.1%), specificity of 77% (95% CI 70.4–82.5%), PPV of 77% (95% CI 70.4–82.5%) and NPV of 95.9% (95% CI 91.4–98.2%) and could correctly classify 282/330 (85.5%, 95% CI 81.2–88.5%) ‘malignant *versus* benign’ lesions with respect to pathology (Table 4).

**Table 4:** Material density parameters of malignant and benign nodules on spectral computed tomography

MD parameters	Malignant (HCC and indeterminate) 147	Benign (regenerative) 183	*p* value
On HAP			
ICD_nodule_ (mg/mL)	19 (16–25)	9 (4–15)	< 0.001
LNR	2.8 (1.8–5.5)	0.8 ( 0.5–1.8)	< 0.001
NIC (mg/mL)	0.2 (0.1–0.2)	0.1 (0.04–0.1)	< 0.001
ICD_liver_ (mg/mL)	6 (3–11)	8 (4–12)	0.002
On PVP			
ICD_nodule_ (mg/mL)	10 (6–14)	13 (8–19)	0.004
LNR	0.6 (0.4– 0.8)	0.6 (0.4–0.8)	0.869
NIC (mg/mL)	0.2 (0.1–0.4)	0.3 (0.2–0.5)	0.003
ICD_liver_ (mg/mL)	16 (12–23)	18 (14–27)	0.007
Difference between HAP and PVP			
ΔICD_nodule_ (mg/mL) Mean ± SD	8.3±11.2	-2.9 ± 9.5	< 0.001

All MD values are presented as median (interquartile range)

*HAP* Hepatic arterial phase, *ICD* Iodine concentration density, *LNR* Lesion-to-normal liver iodine density (LNR = ICD lesion/ICD normal liver), *MD* Material density of iodine, *NIC* Normalised iodine concentration (NIC = ratio of ICD_nodule_/ICD_aorta_), *PVP* Portal venous phase, *SD* Standard deviation, *ΔICD* Delta iodine concentration (ΔICD = ICD_nodule_ HAP minus ICD_nodule_ PVP)

The Mann-Whitney test was applied between the two continuous data (Table 4) and revealed that median ICD_nodule_ HAP was higher (*p < *0.001) for malignant 19 mg/mL (IQR 16–25) *versus* benign 9 mg/mL (IQR 4–15 mg/mL) nodules. LNR was greater (*p* < 0.001) for malignant 2.8 (IQR 1.8–5.5) *versus* 0.8 (IQR 0.5–1.8) for benign nodules on HAP. NIC-HAP was higher (*p* < 0.001) for malignant 0.2 mg/mL (IQR 0.1–0.2) *versus* 0.1 mg/mL (IQR 0.04–0.1) benign lesions, and NIC-PVP was lower (*p* = 0.001) for malignant 0.2 mg/mL (IQR 0.1–0.4) *versus* 0. 3 mg/mL (IQR 0.2–0.5) for benign nodules. ICD_nodule_ PVP was greater (*p* = 0.004) for benign 13 mg/mL (IQR 8–19 mg/mL) *versus* 10 mg/mL (IQR 6–14 mg/mL) for malignant lesions. The ΔICD was significantly higher (*p* < 0.001) for malignant (8.3±11.2 mg/mL) than benign (-2.9 ± 9.5 mg/mL) nodules (Table 4).

All parameters except LNR-PVP which was the same 0.6 (IQR 0.4– 0.8) for both groups (*p* = 0.869) showed statistically significant values (*p* < 0.05) for nodule discrimination

**Fig. 5:**
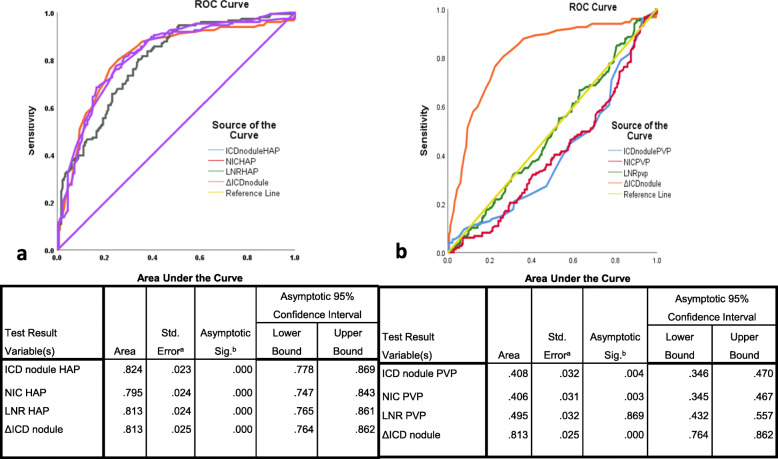
Receiver operating curves of material density parameters with area under curve, sensitivity, and specificity of each parameter as tabulated below. **a** ROC curves plotted on HAP. **b** ROC curves plotted on PVP

For the discrimination of malignant nodules, ICD _nodule_ HAP showed AUC 82.4% (95% CI 77.8–86.9%), cutoff ≥ 15.5 mg/mL (sensitivity 77.4%, specificity 75.3%, *p* < 0.001); LNR-HAP showed AUC 81.3% (95 % CI 76.5–86.1%), cutoff > 1.80 (sensitivity 76%, specificity 75.3%, *p* < 0.001) (Fig. 5). ΔICD demonstrated AUC 81.3% (95% CI 76.4–86.2%) with cutoff >3.5 mg/mL (sensitivity 76%, specificity 78%, *p* < 0.001) (Fig. 5).

The MD parameters on PVP demonstrated AUC between 40 and 50%, sensitivity (50–58%) and specificity (50–60%) as depicted in Fig. 5 along with the cutoff values (Supplementary Table 4).

MD parameters were analysed in both phases to define threshold values for differentiation of *three* cirrhotic nodules (Supplementary Figure 2) since pathology could not be considered as an absolute ground truth in case of dysplastic or regenerative nodules.

Nominal logistic regression showed that ICD_nodule_ HAP > 18.5 mg/mL with odds ratio (OR) 1.22 (95% CI 1.17–1.27) correctly classified HCC in 89% cases, 11.5–18.5mg/mL was consistent for dysplastic nodules with OR 1.17 (95% CI 1.11–1.22) and values < 11.5 mg/mL correctly classified regenerative nodules in 74% with OR 1 (Supplementary Table 2).

## Discussion

The main finding from our study is that MD parameters such as ICD_nodule_ HAP, LNR-HAP, NIC-HAP, ICD_nodule_ PVP, NIC-PVP, and ΔICD provided adequate differentiation between the benign and malignant (including potentially malignant) nodules (all *p* < 0.05). This finding established the feasibility of SCT for diagnosis and classification of cirrhotic nodules. Our results depicted an excellent agreement between the expected enhancement and perfusion pattern of malignant and benign nodules in the cirrhotic liver. We also observed that few MD parameters (ICD_nodule_ HAP, LNR-HAP, ΔICD, and NIC-HAP) showed higher diagnostic accuracy (all AUCs > 80%) than those previously reported (70%) by non-spectral CT [42].

MD parameters on PVP were not as accurate as on HAP. We observed that LNR-PVP did not demonstrate significant demarcation between nodules. This could possibly be due to the unremarkable difference in contrast density of nodules as compared to the underlying liver on PVP, vis-a-vis the contrast divergence usually observed on HAP. Although this observation needs further validation, it implies that more weightage should be given to MD parameters on HAP than on PVP.

Pfeiffer et al. [37] have demonstrated the efficiency of LNR in both HAP and PVP as a better predictor than low (65) keV SCT or MRI for characterising HCC, and Gao et al. [43] demonstrated that LNR-HAP was the best parameter on MD maps to differentiate HCC from other liver nodules in cirrhotic patients.

Our analysis highlighted that ICD_nodule_ HAP is one of the best objective predictors for nodule differentiation. However, ΔICD appears to be the single most dependable marker, to discriminate cirrhotic nodules. This is likely since it incorporates data from both HAP and PVP, yields high diagnostic accuracy (AUC 81%) and demonstrates distinct cutoff values for malignant *versus* benign nodules.

The unique capability of SCT to produce MD-based images which can be used to determine the composition of soft tissues has been extensively explored for oncological imaging [25, 44–47]. Matsui et. al [48] reported that liver nodules differentiate into cancer either via a de novo or a stepwise process where the intranodular hepatic vessels are gradually replaced by tumoural neo-vasculature. This change in nodule vascularity according to its morphological progression can be quantified by measuring their iodine absorption (K*-*edge 33.2 keV of iodine makes it ideal for decomposition) which is an indirect measure of their vascularity [49]. Up to date, there are no clear-cut criteria (from EASL or LI-RADS) for the definition of dysplastic or regenerative nodules [40]. However, it is well known from previous studies that dysplastic and regenerative nodules are distinct entities that are visualised in the cirrhotic liver on different modalities [50]. MD values, which are primarily based on tissue iodine density, are exclusive to the inquired lesion and are independent of observer reading or interpretation. They have the potential to serve as non-invasive biomarkers for liver nodules in cirrhosis.

Cho et. al [51] showed that hepatocarcinogenesis is a time-dependant progression of benign to malignant nodules and this was illustrated in our study, by the presence of regenerative nodules in younger patients with more HCC nodules observed in the older population. Rizal et al. [52] demonstrated that cirrhosis, regardless of aetiology, is more prevalent in men, worldwide. This gender predilection was seen involving all three groups in our study, which was in line with these observations. The median nodule size and AFP also showed a significant increase from the regenerative to the malignant group. This trend indicated a concomitant increment in lesion size and AFP with evolution to malignant disease [53].

The majority of HCC’s on CT were correctly classified in comparison to pathology. Only 3% of the lesions, which demonstrated an atypical enhancement pattern, were misdiagnosed on imaging. These lesions measured < 1 cm on size, which was probably the reason for ambiguous enhancement.

Among the indeterminate nodules categorised on CT, only 10% were confirmed as dysplastic nodules on biopsy. We believe that this was because pathology could not adequately distinguish these nodules distinctly, as shown by previous studies, because low-grade dysplastic nodules either appear normal or show only minimal nuclear atypia and high-grade dysplastic nodules are extremely difficult to distinguish from well-differentiated HCC [9, 10]. We believe that the rest of the 90% of the nodules classified as ‘indeterminate’ on SCT in our study were inadvertently categorised into regenerative or HCC groups on histopathology but could have actually represented dysplastic lesions.

Overall, CT showed a sensitivity and specificity for the diagnosis of malignant nodules of 95% and 77% and for regenerative nodules of 98% and 96%, respectively, to be compared with a previously reported overall CT diagnostic accuracy (approximately 70.5%) for characterisation of cirrhotic nodules [42].

**Fig. 6:**
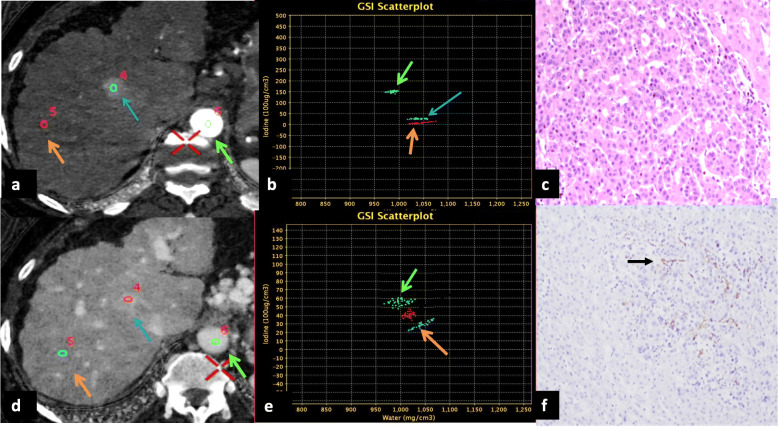
Spectral computed tomography-based material density (MD) map (iodine only image) and scatter plots with histopathological confirmation of an indeterminate nodule. **a** Axial MD map on HAP depicting indeterminate nodule with ROI (blue circle) placed at its centre (blue arrow), ROI (red circle) placed on the background liver parenchyma (orange arrow) and ROI (green circle) within the aorta at the same level (green arrow). **b** Scatter plot demonstrating ICD_nodule_ HAP for nodule (blue arrow) more than the background liver ICD_liver_ HAP (orange arrow) and less than ICD_aorta_ (green arrow) as reference. **c** Histopathology confirmation of nodule, showing features of high-grade dysplasia. **d** Axial MD map acquired on PVP showing ROI (red circle) placed in the centre of the nodule (blue arrow), ROI (blue circle) on the liver parenchyma (orange arrow), and aorta (green circle) at the same level (green arrow). **e** Scatter plot demonstrating ICD_nodule_ PVP (blue arrow) persistently higher than ICD_liver_ PVP (orange arrow). **f** Immunohistochemistry slide showing CD34-positive foci (black bold arrow) in the nodule depicting dysplasia

Subsequently, MD parameters were applied to two nodule groups: malignant (comprising of HCC and potentially malignant/indeterminate lesions) and benign (regenerative) nodules, which demonstrated that all parameters could adequately discriminate the two categories with higher diagnostic accuracy of arterial phase parameters. Thus, SCT seems to yield the potential to be an optimum screening modality for dysplastic nodules and to distinguish them from regenerative nodules (Fig. 6). All nodules with ICD_nodule_ HAP values > 11.5 mg/mL and atypical enhancement pattern could be monitored with a high index of suspicion by the radiologist in tandem with the treating physician for stricter surveillance, till the end point of either the nodule exhibiting classical imaging appearance of HCC or values > 15.5 mg/mL which has been ascertained as a valid cutoff for malignancy with reference to pathology.

Although beyond the scope of this study, long-term studies would be required to evaluate and sequentially follow-up dysplastic nodules on SCT, regardless of pathology correlation, to look for metamorphosis into HCC and to determine MD values based on natural disease progression. In addition, ‘numerical cutoff values’ on SCT would simplify the algorithm for diagnosis of nodules in cirrhosis by facilitating ‘operator independent’, machine-based learning in the future. We feel that extraction of objective MD values will also open the pathway for demarcation of a variety of other benign (*e.g.*, haemangioma, fibronodular hyperplasia) and non-HCC malignant tumours in the liver.

As our objective was to distinguish nodules in cirrhosis using quantitative MD parameters, we have defined three main MD parameters (ICD_nodule_ HAP, LNR-HAP, and ΔICD) and generated cutoff values for differentiation of malignant or potentially malignant and benign nodules in cirrhosis. These are likely to help radiologists gain confidence while reporting ‘lesions in question’ by circumventing, operator-dependency, scanner limitations, technical parameters, contrast-injection protocols and hemodynamic changes etc. Spectral CT appears as an interesting functional tool for characterisation of nodules in the cirrhotic liver and likely to open a cemented pathway to radiomics in the near future. The incorporation of MD parameters as quantitative and objective criteria for the adequate distinction of these nodules would help in triaging patients for follow-up, further evaluation or intervention. Early therapeutic measures for potentially malignant lesions will open a new window of opportunities in overall disease survival and prognosis.

There were limitations of our study: lack of an adequate number of dysplastic lesions on histopathology for reference; exclusion of other incidental lesions such as haemangiomas; lipomas, complex cysts; fibronodular hyperplasia; or hepatic adenoma from our study cohort. We also excluded non-HCC tumours and most of the atypical HCC or variants such as combined HCC-cholangiocarcinoma, during selection of the patient population. We did not use LI-RADS for initial nodule characterisation in addition to the EASL criteria to designate nodules into more than three categories. In the future, studies are required to assess other hepatic focal lesions such as cysts, mimics of HCC and non-HCC malignancies with quantitative MD parameters for a comprehensive approach. LI-RADS algorithm may also be used in conjunction with MD values for each nodule category in liver cirrhosis.

In conclusion, spectral CT-generated MD parameters, namely ICD_nodule_ HAP, LNR-HAP, and ΔICD, provided a viable diagnostic tool for differentiating malignant or potentially malignant from benign nodules in cirrhotic patients. Such parameters may represent an add-on diagnostic tool for hepatic nodule characterisation.

## Supplementary Information

**Additional file 1: Supplementary Table 1.** Enhancement pattern of nodules (n = 330) based on size on computed tomography. **Supplementary Table 2.** Nominal logistic regression analysis for MD parameter ICD _nodule_ HAP. **Supplementary Table 3.** Correlation of CT and histopathology diagnosis of nodules with respect to size. **Supplementary Table 4.** Diagnostic performance of MD parameters on SCT to distinguish malignant *versus* benign nodules. **Supplementary Figure 1.** Canonical scores plot representing all cirrhotic nodules in the study. Canonical scores plot to depict overlap of the indeterminate nodules (depicted as red circular area) into either category of HCC and regenerative nodules (green circular plots). **Supplementary Figure 2.** Classification and regression tree (CART) analysis of ICD _nodule_ HAP.

## Data Availability

All data generated or analysed during this study are included in this manuscript and its supplementary information files.

## Abbreviations

AFP: Alpha-fetoprotein
AUC: Area under the curve
CI: Confidence interval
EASL: European Association for the Study of the Liver
HAP: Hepatic arterial phase
HCC: Hepatocellular carcinoma
ICD: Iodine concentration density
LI-RADS: Liver Imaging Reporting and Data System
LNR: Lesion-to-normal ratio
MD: Material density
NIC: Normalised iodine concentration
PVP: Portal venous phase
ROI: Region of interest
SCT: Spectral computed tomography
ΔICD: Delta in iodine concentration density
