# Supplementary material for: Material decomposition using iodine quantification on spectral CT for characterising nodules in the cirrhotic liver: a retrospective study
Source: Eur Radiol Exp. 2021 May 28;5:22. doi: 10.1186/s41747-021-00220-6 (PMC8160046; doi:10.1186/s41747-021-00220-6)

**ELECTRONIC SUPPLEMENTARY MATERIAL**

**Tables**

**Supplementary Table 1** Enhancement pattern of nodules (n=330) based on size on computed tomography

| Nodule size (total number) | Phase of enhancement | | Enhancement Pattern | HCC  133  (%) | Intermediate nodule  50  (%) | Regenerative nodule  147  (%) | Total nodules  330  (%) |
| --- | --- | --- | --- | --- | --- | --- | --- |
| <1cm  (29) | | HAP | Hypervascular | 0  – | 2  4 | 1  0.7 | 3  0.9 |
|  | |  | Hypovascular | 0  – | 0  – | 22  14.9 | 22  6.7 |
|  | |  | Mild enhancement | 0  – | 0  – | 4  2.7 | 4  1.2 |
| 1-2cm  (198) | | HAP | Hypervascular | 33  24.8 | 20  40 | 0  – | 53  16 |
|  | |  | Hypovascular | 2  1.5 | 10  20 | 105  31.2 | 117  35.5 |
|  | |  | Mild enhancement | 13  9.7 | 14  28 | 1  0.7 | 28  8.5 |
| >2cm  (103) | | HAP | Hypervascular | 65  48.9 | 2  4 | 1  0.7 | 68  20.6 |
|  | |  | Hypovascular | 6  4.5 | 1  2 | 13  8.8 | 20  6 |
|  | |  | Mild enhancement | 14  10.5 | 1  2 | 0  – | 15  4.5 |
| <1cm  (29) | | PVP | Enhancement present | 0  – | 1  2 | 24  16.3 | 25  7.6 |
|  | |  | No enhancement | 0  – | 1  2 | 3  2 | 4  1.2 |
| 1-2 cm  (98) | | PVP | enhancement present | 47  35 | 41  82 | 105  71.4 | 193  58.5 |
|  | |  | No enhancement | 1  0.7 | 3  6 | 1  0.7 | 5  1.5 |
| >2 cm  (103) | | PVP | enhancement present | 84  63.1 | 4  8 | 14  9.5 | 102  30.9 |
|  | |  | No enhancement | 1  0.7 | 0  – | 0  – | 1  0.3 |
| <1cm  (29) | | Washout present | | 0  – | 2  4 | 1  0.68 | 3  0.9 |
| 1-2cm  (198) | | Washout present | | 46  34.6 | 36  72 | 0  – | 82  24.8 |
| >2cm  (103) | | Washout present | | 79  59.4 | 2  4 | 0  – | 81  24.5 |

All nodule sizes were considered as the largest measured diameter on CT

*HAP* hepatic arterial phase, *PVP* portal venous phase, *HCC* hepatocellular carcinoma

**Supplementary Table 2** Nominal logistic regression analysis for MD parameter ICD _nodule_ HAP

| Nodules | | Sig. | Odds Ratio | 95% Confidence Interval for Exp(B) | |  |
| --- | --- | --- | --- | --- | --- | --- |
|  |  |  |  | Lower Bound | Upper Bound |  |
| HCC | Intercept | 0.000 |  |  |  |  |
|  | ICD nodule HAP | 0.000 | 1.217 | 1.166 | 1.271 |  |
| dysplastic | Intercept | 0.000 |  |  |  |  |
|  | ICD nodule HAP | 0.000 | 1.165 | 1.111 | 1.222 |  |
|  |  |  |  |  |  |  |
|  |  |  |  |  |  |  |

Odds Ratio for regenerative nodule=1

Nominal logistic regression analysis showing ICD _nodule_ HAP > 18.5 mg/ml with Odds ratio (OR) 1.22 (95% CI 1.17–1.27) correctly classified HCC in 89% cases

ICD _nodule_ HAP of 11.5–18.5mg/ ml was connotative for dysplastic nodules with OR 1.17 (95% CI 1.11–1.22)

ICD _nodule_ HAP < 11.5 mg/ml correctly classified regenerative nodules in 74% with Odds Ratio for regenerative nodule=1

**Supplementary Table 3** Correlation of CT and histopathology diagnosis of nodules with respect to size

| Size on CT | Imaging diagnosis | HCC  136  (41.2%) | Dysplastic  11  (3.3%) | Regenerative  183  (55.5%) | Total nodules  330 |
| --- | --- | --- | --- | --- | --- |
| <1 cm  Total nodules 29 | HCC | 0 | 0 | 0 | 0 |
|  | Indeterminate | 0 | 0 | 2 | 2 |
|  | Regenerative  PPV (%)  Sensitivity (%) | 1  –  – | 0  –  – | 26  96.3  92.9 | 27  –  – |
|  | Total nodules on CT | 1  (3.4) | 0  – | 28  (96.5) | 29  – |
| 1-2 cm  Total nodules 198 | HCC  PPV (%)  Sensitivity (%) | 45  93.8  90 | 0  –  – | 3  –  – | 48  –  – |
|  | Indeterminate  PPV (%)  Sensitivity (%) | 3  –  – | 4  9.1  100 | 37  –  – | 44  –  – |
|  | Regenerative, n  PPV (%)  Sensitivity (%) | 2  –  – | 0  –  – | 104  98.1  72.2 | 106  –  – |
|  | Total nodules on CT | 50  (25) | 4  (2) | 144  (72.7) | 198 |
| >2 cm  Total nodules 103 | HCC  PPV (%)  Sensitivity (%) | 84  98.8  98.8 | 1  –  – | 0  –  – | 85  –  – |
|  | Indeterminate  PPV (%)  Sensitivity (%) | 1  –  – | 3  75  42.9 | 0  –  – | 4  –  – |
|  | Regenerative  PPV (%)  Sensitivity (%) | 0  –  – | 3  –  – | 11  78.6  100 | 14  –  – |
|  | Total nodules on CT | 85  (82.5) | 7  (6.8) | 11  (10.7) | 103  – |

*CT* computed tomography, *HCC* hepatocellular carcinoma, *PPV* positive predictive value

**Supplementary Table 4** Diagnostic performance of MD parameters on SCT to distinguish malignant versus benign nodules

| CT Phase | Parameter | Area under the curve (95% CI) | Asympt-otic Sig. ^b^ | Cutoff  values | Sensitivity  (%) | Specificity  (%) |
| --- | --- | --- | --- | --- | --- | --- |
| HAP | ICD nodule (mg/ml) | 82.4 (77.8–86.9) | 0.000 | > 15.5 | 77.4 | 75.3 |
|  | NIC (mg/ml) | 79.5 (74.7–84.3) | 0.000 | >1.24 | 71.2 | 70.3 |
|  | LNR | 81.3 (76.5–86.1) | 0.000 | >1.80 | 76 | 75.3 |
| PVP | ICD nodule (mg/ml) | 40.8 (34.6–47.0) | 0.004 | <11.50 | 54.1 | 61.2 |
|  | NIC (mg/ml) | 40.6 (34.5–46.7) | 0.003 | <0.26 | 57.9 | 55.1 |
|  | LNR | 49.5 (43.2–55.7) | 0.869 | <0.64 | 50.3 | 50 |
| HAP-PVP | Δ ICD (mg/ml) | 81.3 (76.4–86.2) | 0.000 | >3.50 | 76 | 78 |

*SCT* Spectral computed tomography*, HAP* hepatic arterial phase, *ICD* iodine concentration density, *LNR* lesion to normal liver iodine density, *NIC* normalized iodine concentration, *Δ ICD* delta iodine concentration

**Figures**

Supplementary Fig. 1 Canonical scores plot representing all cirrhotic nodules in the study

Canonical scores plot to depict overlap of the indeterminate nodules (depicted as red circular area) into either category of HCC and regenerative nodules (green circular plots)

Supplementary Fig. 2 Classification and regression tree (CART) analysis of ICD _nodule_ HAP


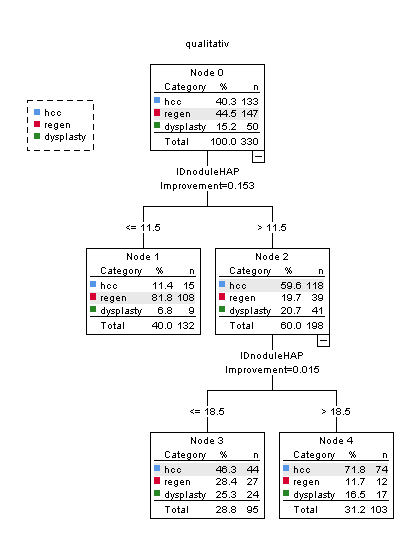

Supplement: Supplementary file 1 — Additional file 1: Supplementary Table 1. Enhancement pattern of nodules (n = 330) based on size on computed tomography. Supplementary Table 2. Nominal logistic regression analysis for MD parameter ICD nodule HAP. Supplementary Table 3. Correlation of CT and histopathology diagnosis of nodules with respect to size. Supplementary Table 4. Diagnostic performance of MD parameters on SCT to distinguish malignant versus benign nodules. Supplementary Figure 1. Canonical scores plot representing all cirrhotic nodules in the study. Canonical scores plot to depict overlap of the indeterminate nodules (depicted as red circular area) into either category of HCC and regenerative nodules (green circular plots). Supplementary Figure 2. Classification and regression tree (CART) analysis of ICD nodule HAP. [file 41747_2021_220_MOESM1_ESM.docx]
